# Absence of calretinin protein expression in malignant mesotheliomas from asbestos-exposed NF2^+/−^ mice and mouse mesothelioma cell lines from various mouse strains

**DOI:** 10.1186/s40364-018-0132-0

**Published:** 2018-06-06

**Authors:** Walter Blum, Thomas Henzi, Hugues-Etienne Châtel-Soulet, Laszlo Pecze, Janine Wörthmüller Rodriguez, Bart Vrugt, Beat Schwaller

**Affiliations:** 1 Anatomy, Section of Medicine, University of Fribourg, Route Albert-Gockel 1, CH-1700 Fribourg, Switzerland; 2 Institute of Pathology and Molecular Pathology, University Hospital Zurich, Zurich, Switzerland

**Keywords:** Malignant mesothelioma, Calretinin, Calb2, C57Bl/6J, NF2^+/−^ mice, Asbestos

## Abstract

**Background:**

Calretinin is the most widespread positive marker for the immunohistochemical identification of malignant mesothelioma (MM) and was proposed to serve as a blood-based biomarker. Functionally, evidence has accumulated that calretinin might be implicated in MM tumorigenesis. We aimed to identify calretinin (CR; *Calb2*) in murine MM and reactive mesothelial cells in granuloma from asbestos-exposed NF2^+/−^ mice, a line heterozygous for the tumor suppressor merlin (NF2), used as a mouse MM model. Additionally, we sought to ascertain the presence of calretinin in MM cell lines from other mouse strains. We also intended to investigate the role of calretinin in mesotheliomagenesis by comparing the survival of asbestos-exposed NF2^+/−^ and NF2^+/-^CR^−/−^ mice.

**Methods:**

NF2^+/−^ and NF2^+/-^CR^−/−^ mice, both lines on a C57Bl/6J background, were exposed to asbestos following an established protocol. Tumor histology and asbestos-induced mortality were assessed. MM and granuloma from NF2^+/−^ mice were analyzed with immunohistochemical methods for calretinin expression. Levels of *Calb2* mRNA and calretinin expression in tumors and MM cell lines of various mouse strains were determined by RT-qPCR and Western blot analysis, respectively.

**Results:**

No expression of calretinin at the protein level was detected, neither in MM from NF2^+/−^ mice, NF2^+/−^ MM-derived cell lines nor immortalized mesothelial cells of mouse origin. At the mRNA level we detected *Calb2* expression in MM cell lines from different mouse strains. Survival of NF2^+/−^ and NF2^+/-^CR^−/−^ mice exposed to asbestos showed no significant difference in a log-rank (Kaplan-Meier) comparison.

**Conclusions:**

The concomitant determination of calretinin and mesothelin blood levels has been proposed for early detection of human MM. Mouse MM models based on asbestos exposure are assumed to yield helpful information on the time course of appearance of mesothelin and calretinin in the blood of asbestos-treated mice determining the earliest time point for interventions. However, the observed absence of calretinin in MM from NF2^+/−^ mice and derived cell lines, as well as from MM cells from Balb/c and C3H mice likely precludes the use of calretinin as a biomarker for mouse MM. The results also indicate possible species differences with respect to an involvement of calretinin in the formation of MM.

## Background

Malignant mesothelioma (MM) is an extremely aggressive neoplasm linked to asbestos exposure [1]. Median survival is very short (9–12 months), likely linked to diagnosis at late stages of the disease. Correct pathological diagnosis is of outmost importance and calretinin has been described as one of the most sensitive and selective positive markers for human MM [2, 3]. Calretinin (CR; human gene symbol: *CALB2*) is a calcium-binding protein of the EF-hand family with several reported functions, see [4]. Together with mesothelin, the detection of CR in blood samples by ELISA has been suggested as a potential predictor to detect MM at earlier stages [5, 6]. Other discussed MM biomarkers include soluble mesothelin-related peptides, megakaryocyte potentiating factor, osteopontin, fibulin-3 and high mobility group protein B1 (HMGB1) [7]. However, the time course of appearance of these biomarkers during human MM pathogenesis is currently unknown. Animal models (mostly mice) are widely used as models to address such questions.

Common genomic alterations and similar genomic profiles, e.g. loss of tumor suppressors (NF2, LATS2, BAP1) in murine and human MM emphasize the relevance of mouse model systems to study mesotheliomagenesis [8] and to possibly validate suggested MM biomarkers. Such genetic mouse MM models include mice heterozygous for NF2, one of the most often altered tumor suppressor genes with up to 44% alterations in human MM cell lines [9, 10]. Asbestos-exposed NF2^+/−^ mice were reported to be an important murine model recapitulating many molecular features of human MM and to be relevant for further investigations on MM pathogenesis mechanisms, as well as for preclinical testing of putative novel therapeutic approaches [11, 12]. The aim of this study was to investigate the expression of calretinin in I) murine MM originating from NF2^+/−^ mice; II) MM cell lines derived from these mice and III) murine MM cell lines from strains other than C57Bl/6J, the genetic background of the NF2^+/−^ mice. In addition, to investigate any potential role for CR in murine MM formation we assessed whether overall survival differed between asbestos-exposed NF2^+/-^ mice with or without a functional *Calb2* gene.

## Methods

### Animal studies

129S2/SvPas Nf2+/− mice (strain name: B6;129S2-*Nf2*^*tm1Tyj*^/J; JAX stock #008190) were backcrossed for ≥6 generations to wild-type C57Bl/6J (C57) mice to obtain a C57-NF2^+/−^ mouse line. These mice were crossed with C57-CR^−/−^ mice [13] to give rise to the genotype C57-NF2^+/-^CR^−/−^; animals were genotyped as described previously [14]. Mice, 6–8 weeks of age, were injected intraperitoneally (i.p.) with 400 μg of crocidolite suspended in 500 μL PBS every 3 weeks for a total of eight rounds of injection (i.e., a total of 3.2 mg of crocidolite per mouse as described before [12]). Unio Internationale Contra Cancrum (UICC) grade crocidolite asbestos was obtained from SPI Supplies (West Chester, PA). For survival analysis, mice were kept for a maximum of 33 months.

Four NOD/SCID gamma mice were injected i.p. with a suspension (200 μl PBS) of FACS-sorted ZL55-SO^high^ cells (100,000/mouse). These genetically-modified human MM cells are identified by their increased endogenous expression of the stem cell markers SOX2 and OCT4 driving the reporter eGFP, the latter allowing for the isolation of this small (< 5%) subpopulation [15]. ZL55-SO^high^ cells show an increased tumor-initiating capacity in vivo compared to the parental ZL55 cells. Mice were sacrificed after 5 weeks and tissue was fixed in 4% paraformaldehyde. Histopathological diagnosis and analyses were performed on paraffin-embedded samples as described before [16]. All animal experiments were performed with the permission of the local animal care committee (Canton of Fribourg, Switzerland) and according to the present Swiss law and the European Communities Council Directive 86/609/EEC.

### Western Blotting

Proteins from murine MM cell lines and from C57-NF2^+/−^ tumor samples were prepared as described before [16]. Briefly, protein extracts from MM cell lines (AB12, AK7, RN5 [16], RN29) were separated by SDS-PAGE (10% gel) and transferred onto a nitrocellulose membrane. The membrane was incubated with primary antibody against calretinin (rabbit polyclonal CR7696 [17]), previously available from SWANT, Marly, Switzerland at a dilution of 1:10,000. Secondary biotinylated anti-rabbit IgG at a dilution of 1:10,000 for 2 h at RT was used. For signal detection the ABC system (Vectastain, Vector Laboratories, Burlingame, CA) was applied and chemiluminescent signals were revealed with the HRP substrate (Millipore, Luminata Forte); signals were detected with the Western blot reader (FluorChem E System, Bucher Biotec, Basel, Switzerland) as previously described [18].

### Histological analysis

Tumor samples were fixed in 4% paraformaldehyde in PBS, pH 7.4, dehydrated and embedded in paraffin followed by sectioning with a microtome. Tumor samples were pathologically evaluated using hematoxylin/eosin or Goldner-stained sections. For immunohistochemistry, sections were deparaffinized and incubated in antigen-retrieval solution using sodium citrate, pH 6, and processed as described before [19]. Primary antibodies (CR7696, CR7697 or CR7699/4; SWANT, Marly, Switzerland) were used at a 1:500 dilution with overnight incubation at 4 °C.

### Semi-quantitative RT-PCR of *Calb2* mRNA levels

The expression of murine *Calb2* mRNA was detected by semi-quantitative RT-PCR. Murine mesothelioma cell lines were cultured under standard conditions [16] and total RNA extracted with PeqGold Trifast (Axonlab, Le Mont-sur-Lausanne, Switzerland) following the manufacturer’s protocol. cDNA was prepared from 500 ng total RNA using the QuantiTect Reverse Transcription kit (Qiagen, Stockach, Germany) by following manufacturer’s instructions. As housekeeping gene the ribosomal protein 13S was used and *Calb2* expression was assessed using the following primers: Calb2 -FW: 5′-GAA ATG GGT ACA TTG AAG GTA-3′, Calb2 RV 5’-CCA TCT GAG TTC TTC TTA TCA TAC 5′, RPS-13 FW 5’CGA AAG CAT CTT GAG AGG AAC A 3′, RPS-13 RV 5’-TCG AGC CAA ACG GTG AAT C-3′.

### Statistical analysis

GraphPad Prism version 7 and R statistical software were used to perform the log-rank test (Mantel-Cox test) on the Kaplan-Meier survival plot for comparison.

## Results

**Fig. 1:**
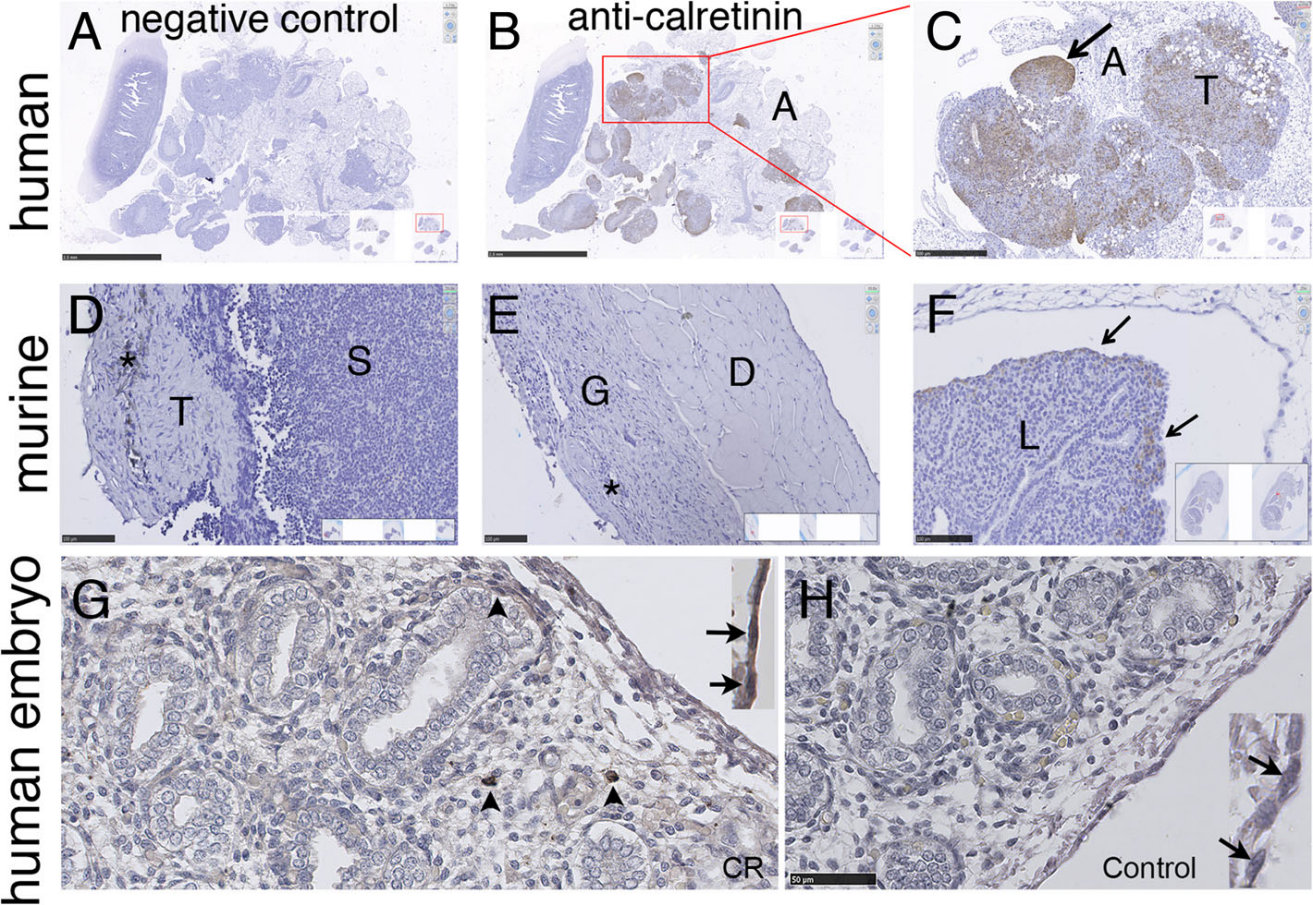
Immunohistochemical analysis of human and mouse MM and embryonic mouse and human lung. ZL55-SO^high^ cells were injected i.p. into NOD/SCID gamma mice and tumor cells show strong expression of CR (**b**; higher magnification inset in **c**), arrow pointing towards a strong CR-positive tumor nodule. The negative control (parallel section) is shown in (**a**). **d**) Representative example of a mouse MM showing absence of CR expression, as well as in another example of an asbestos-induced lymphocytic and granulomatous inflammation (**e**) of the parietal pleura. **f**) In the developing lung of a mouse, CR protein is already expressed in the visceral pleura at day 10 (arrows) as reported before [14]. **g**) Expression of CR in the mesothelial cell layer (arrows) and in scattered stromal (mesenchymal) cells of the developing human lung (arrowheads). **h**) Negative control on parallel section incubated without primary CR antibody. The inset shows CR-negative mesothelial cells (arrows) at 2-fold higher magnification. The few round lightly brownish-stained cells are erythrocytes, where the endogenous peroxidase activity was not completely blocked. Abbreviations: A adipose tissue; T tumor; S stroma; G granulomatous inflammation; D pleura parietalis adjacent to the diaphragm; L lung. Scale bars: A and B: 2.5 mm, C: 500 μm. D-F: 100 μm; G,H: 50 μm; insets with layer of mesothelial cells: 2-fold magnification compared to G,H

The repetitive injection of crocidolite in C57-NF2^+/-^ mice resulted in approximately 10% of confirmed murine mesothelioma cases and in a multitude of granuloma formation in treated animals [12]. Granuloma were described to be rich in reactive mesothelial cells and human reactive mesothelial cells were previously shown to be strongly positive for CR [20]. Human ZL55 MM cells injected i.p. into NOD/SCID gamma mice were used as a positive control. ZL55-derived tumors showed strong calretinin IHC staining, clearly a differentiating marker for the tumor nodules located in connective tissue often consisting of CR-negative nodules of adipose tissue (Fig. 1a-c). None of the C57-NF2^+/−^ mice-derived MM cells and the reactive mesothelial cells on the peritoneal side of the diaphragm from the same mice showed CR expression in the IHC analysis, representative examples are shown in Fig. 1d-e. As previously described [14], the developing lung of the murine embryo shows staining for CR in the visceral mesothelial layer and in a fraction of mesenchymal cells (Fig. 1f), even though staining is weaker compared to human mesothelioma [14]. Also in the developing human lung, cells of the visceral pleura, i.e. the outermost mesothelial cell layer, as well as a subpopulation of stromal (mesenchymal) cells stained positive for CR, in particular evident as brown-stained cytoplasm in mesothelial cells in Fig. 1g (inset; arrows). Few stromal cells in the mesenchyme showed very strong staining (Fig. 1g, arrowheads). Note the absence of specific CR staining in the developing epithelial cells of the lung. A control section stained without primary CR antibody was negative (Fig. 1h) including the single layer of mesothelial cells (inset; arrows).

**Fig. 2:**
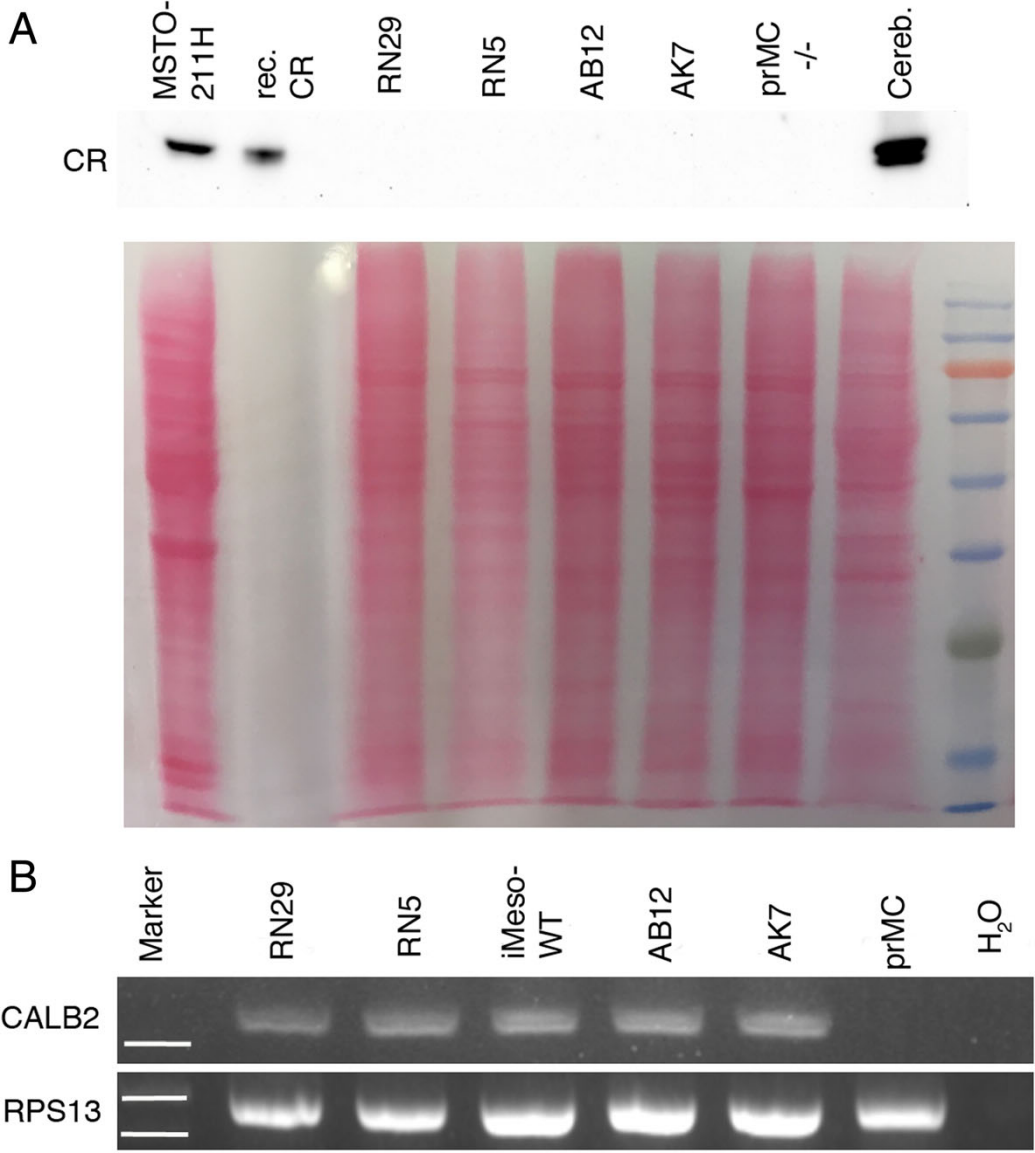
Western Blot analysis for calretinin and RT-PCR for the detection of *Calb2* mRNA. **a**) Western blot signals (upper panel) and the corresponding Ponceau Red-stained membrane (lower panel). As positive control, 10 ng of purified human recombinant CR was used. Sizes (molecular masses) of the marker proteins in the right lane are (from top to bottom): 130, 100, 75 (red), 63, 48, 35, 28 (green), and 17 kDa. The specific signal for CR runs just slightly above the green marker protein at approximately 30 kDa. In MSTO-211H cell extracts, a strong signal at the same position as purified CR indicates the presence of CR in these cells. All mouse samples from the MM cell lines RN29, RN5 and AK7 (all from C57Bl/6 J mice), AB12 (Balb/c) are negative, as well as the negative control prMC from a CR^−/−^ mouse (prMC−/−). Mouse cerebellum extract (most right) shows a strong signal for CR. **b**) Positive signals for *Calb2* mRNA were detected in RN29, RN5, AB12, AK7 and iMeso-WT cells; no signal was present in samples from freshly isolated C57 mouse prMC and in the negative control (H_2_O). RPS13 was used as housekeeping gene

In order to confirm the IHC results we performed Western blot analysis to detect CR expression in murine MM cell lines. Even when loading ≥50 μg of protein no specific CR signal at approximately 30 kDa was detected in all mouse cell lines (Fig. 2a) and in immortalized mesothelial cells from a C57 mouse (iMeso-WT1 [16, 21]; not shown), while a signal was evident in extracts from the human MM cell line MSTO-211H, as well as in mouse cerebellum, described to be rich in CR [22] (Fig. 2a). A protein extract from isolated primary mesothelial cells of a C57-CR^−/−^ mouse served as negative control. *Calb2* was detected at the mRNA level in all tested murine MM cell lines and in mesothelial iMeso-WT1 cells (Fig. 2b), but not in mouse primary mesothelial cells maintained in culture for 24 h, the latter as reported before [14].

In human MM, calretinin is considered to be the most sensitive and selective marker for the diagnosis, in particular of the epithelioid and the epithelioid part of the biphasic MM type [3]. Furthermore, its essential function in vitro in human MM cell lines [18], as well as its function in protection from asbestos-induced cytotoxicity has been demonstrated before [23]. While the clinical relevance of calretinin IHC in the diagnosis of human MM is undisputed, the putative role of calretinin in the development of MM in vivo has remained elusive. Thus, mouse survival after asbestos exposure was tested in a well-studied MM mouse model, i.e. in mice heterozygous for the tumor suppressor NF2 [11] either wild-type (C57-NF2^+/−^) or null-mutant (C57-NF2^+/-^CR^−/−^) for the *Calb2* gene. Long-term survival data were analyzed by the standard Kaplan-Meyer method and differences between groups were assessed using a log-rank test. Mice (48 in total) of the two genotypes were treated with asbestos (NF2^+/-^CR^+/+^, *n* = 18; NF2^+/-^CR^−/−^; *n* = 30). A total of 17 (94%) mice (2 with diagnosed MM) died in the NF2^+/-^CR^+/+^ group (censored, *n* = 1) and 28 (93%; 3 with diagnosed MM) died in the NF2^+/-^CR^−/−^ group (censored, *n* = 2) until the end of the long-term follow up in the 2 cohorts. Kaplan-Meier curves were similar in both groups. Median survival was 14.22 months (95% confidence interval: 9.23–19.07) in mice with a functional *Calb2* gene (NF2^+/-^CR^+/+^) and 14.63 months (95% confidence interval 13.4–17.3) in mice with a non-functional *Calb2* gene (NF2^+/-^CR^−/−^). A log-rank test indicated no significant difference in survival (*p* = 0.7345). The log-rank hazard ratio (NF2^+/-^CR^+/+^ used as reference) was 0.90 (95% confidence interval 0.49–1.66). This indicates that mice with a functional *Calb2* gene have a marginally higher, however insignificant, risk of death after asbestos exposure.

## Discussion

Our results indicate that MM derived from asbestos-exposed C57-NF2^+/−^ mice do not express calretinin protein levels detectable by either IHC or Western blot analysis. No positive Western blot signal was observed in RN5 and RN29 MM cells derived from MM tissue of the same asbestos-exposed C57-NF2^+/−^ mouse strain; yet, RN5 and RN29 cells express *Calb2* mRNA. Likewise, well-established murine MM cell lines AK7 [24] also from C57 mice, and AB12 cells from Balb/c mice were negative for CR protein expression and positive for *Calb2* mRNA. Based on the previous observation of a very strong positive correlation between CR protein and *CALB2* mRNA levels in 13 human MM cell lines, i.e. regulation mostly at the transcriptional level (see Fig. 1c in [25]), we assume levels of *Calb2* mRNA to be low in mouse MM cell lines and MM tissue with no detectable CR protein levels. However, species differences in CR regulation may not be entirely excluded. Thus, in contrast to human MM, where calretinin is considered as a positive marker, we did not detect calretinin at the protein level in MM and derived cell lines of mouse origin. Even human sarcomatoid MM cell lines (e.g. ZL34, ONE58), as well as biphasic MM (e.g. MSTO-211H, Mero83) show a clear and in few cases relatively strong CR expression evidenced by Western blot analysis [18, 25]. Our negative CR IHC results are in contrast to previous reports showing positive CR IHC staining in murine pleural MM samples. In toxicology studies, Yokohira et al. reported positive CR immunostaining of the lung surface in female A/J mice exposed to 4-(methylnitrosamino)-1-(3-pyridyl)-1-butanone (NNK) [26] and to potassium octatitanate (TISMO) fibers [27]. In a later study, also mice from additional strains (C3H/HeN, ICR and C57BL/6), exposed to TISMO showed a thickening of the visceral pleura due to mesothelial cell infiltration with part of the cells showing strong CR immunoreactivity (Fig. 3 in [28]). The differences between previous results and the current ones with respect to CR IHC might be several-fold. IHC results might be related to differences in I) the materials used to induce changes in the tunica serosa (NNK and TIMSO fibers vs. crocidolite), II) the site of injection (pleura vs. peritoneum) and III) the CR antibodies used. With respect to strain differences, Yokohira et al. also reported positive CR immunoreactivity in C57Bl/6 mice (Fig. 3 in [28]), while our tissue samples and cell lines (RN5, RN29, AK7) derived from C57Bl/6 mice were negative for CR. Of note, in the same study by Yokohira et al., weak CR positivity was also detectable in the healthy lung tissue, especially in C57Bl/6 J mice [28], while lung tissue was always completely negative in our study, in line with many studies reporting that normal lung tissue is considered CR-negative. In addition, the specificity of the CR antibody to unequivocally detect mouse CR was not reported in the previous studies, while we have additionally confirmed the specificity of our CR antibodies by Western blotting.

**Fig. 3:**
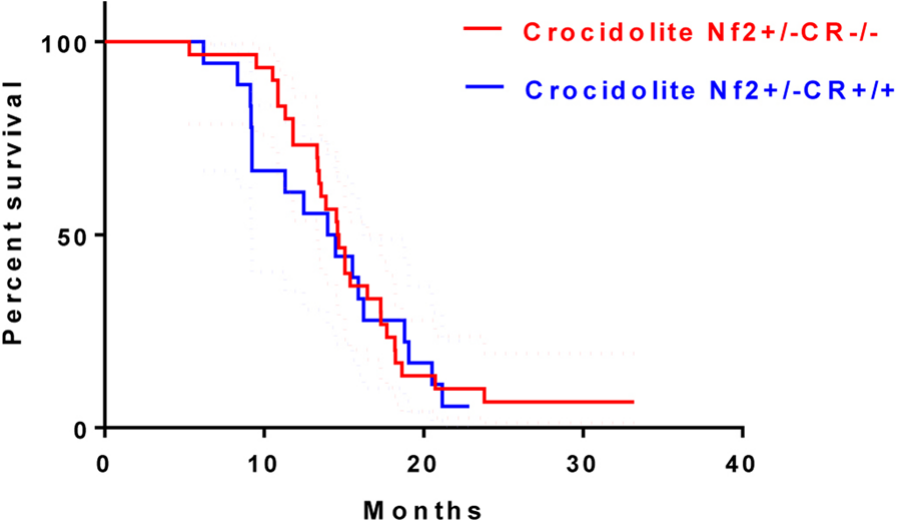
Kaplan-Meier survival analysis. C57-NF2^+/-^CR^+/+^ (blue line; *n* = 18) and C57-NF2^+/-^CR^−/−^ (red line; *n* = 30) mice were injected with 8 × 400 μg of crocidolite. The survival curves of the 2 groups (all mice) are statistically not different (log-rank test, *p* = 0.7345)

A role for calretinin in the development of human MM had been suggested based on in vitro studies. Induced expression of SV40 early gene products augments CR expression in the human mesothelial cell line MeT-5A, which leads to increased resistance to asbestos-mediated acute cytotoxicity, an effect mediated by the AKT/PI3K pathway [23]. Inversely, down-regulation of CR decreases the viability and proliferation of human MM cells in vitro, most evident in epithelioid and biphasic cell lines, but to a lesser extent also in MM cell lines with prevalent sarcomatoid morphology [18]. Moreover, primary mesothelial cells from CR^−/−^ mice are characterized by a lower proliferation rate and are slower to close a scratch evidenced in a 2D-wound assay compared to primary mesothelial cells from wild-type mice [14]. This phenotype disappears, if CR is overexpressed in CR^−/−^ prMC. Based on these results we hypothesized to possibly observe a genotype-dependent effect in the survival of in vivo asbestos-exposed C57-NF2^+/-^CR^−/−^ compared to mice with a functional *Calb2* gene (C57-NF2^+/−^ mice). However, survival of mice in both groups was not different. Of note, in our survival analysis all mice were included, not only the ones with a diagnosed bona fide MM. Given the low penetrance in our model, as reported before (≈10% in C57-NF2^+/−^ mice [12]), i.e. 3/30 in the C57-NF2^+/-^CR^−/−^ mouse group and 2/18 in the C57-NF2^+/-^CR^+/+^ mouse group in this study, the small number of confined MM cases doesn’t allow to directly link *Calb2* genotype to MM-caused mortality. Thus, including all mice might have masked some subtle *Calb2* genotype-dependent differences that are only observable in mice with a diagnosed MM. However, the deaths in both groups were asbestos exposure-dependent, since unexposed mice of both genotypes had a much higher median survival (control C57-NF2^+/-^CR^+/+^ mice) of 29.13 months (95% confidence interval: 5.80–32.93, log-rank test: *p* = 0.0008).

This leads to several different hypotheses. I) The heavy asbestos exposure by direct intraperitoneal fiber injection resulting in relatively short latency for MM development in mice (around 1 year in mice compared to 20–40 years in humans) leads to other routes of MM formation in human and mice. This is partially supported by the observation of mostly sarcomatoid and biphasic MM in mice compared to human, where the largest number of MM cases is of the epithelioid type. However, frequency of gene loci deletions containing genes likely involved in MM formation were reported to be similar in samples from human MM and asbestos-exposed NF2^*+/−*^ mice including *CDKN2A*, *CDKN2B*, *CHD5*, *TP53*, and *NF2* [8]. In contrast, 14/15 cell lines derived from asbestos-injected mice of several mouse strains showed homologous deletions of the *CDKN2A* locus, but no chromosomal losses in either the *BAP1* and *NF2* regions and moreover no mutations in these 2 genes as well as in *LATS2*, commonly mutated in human MM [29]; this might again hint towards species differences in the pathways leading to MM II). Although CR expression is prevalent during both mouse [14] and human lung embryonic development (Fig. 1g, h), CR re-appearance at levels detectable by Western blot analysis selectively in human MM might point towards different (possibly none) functions in the process of murine mesotheliomagenesis compared to the formation of MM in humans. Of note, the presumed role of CR in human MM development is currently based on in vitro studies with human MM cell lines. III) The low CR levels conceivably present in mouse MM (below the detection limit of IHC or Western blot analysis used in this study) still might be sufficient to exert a role, e.g. as a signaling molecule during mouse MM development. IV) Various *Calb2* mRNA transcripts, in part derived from alternative splicing (so far detected only in human MM), might have a function on their own as recently proposed [30] and thus *Calb2* transcripts might possibly be implicated in murine MM tumorigenesis. All of the above points need to be considered, when using mouse MM models with the aim to possibly find strategies that might be applied for developing novel human therapeutic approaches. This is particularly the case when using mouse models aimed at exploiting the putative role of CR in the pathogenesis of human MM. In such a case, xenograft models with human CR-positive MM cells might be considered as the current method of choice. With respect to validating CR as a biomarker for early detection of human MM (currently less than 5% are diagnosed at an early stage 1A [31]), one needs to be aware that CR is unlikely of any predictive value in mouse MM models. Even if CR is expressed at very low protein levels in mouse MM, CR plasma levels are most probably below the detection limit of even most sensitive ELISA techniques.

## Conclusions

Our results indicate the absence of CR (at least below the threshold of Western blot detection) in mouse MM cell lines, in line with negative IHC staining in asbestos-induced mouse MM tissue. These findings essentially rule out the use of CR as a putative biomarker for early detection of MM in mouse models, while this may well be appropriate for human MM. Together with other divergent results in human and mouse MM, e.g. with respect of MM histotype or commonly mutated genes, it requires careful consideration, when translating findings obtained in one species to another.

## Abbreviations

C57: C57Bl/6J
CR: Calretinin
HMGB1: High mobility group B1 protein
MM: Malignant mesothelioma
NF2: Neurofibromatosis 2
